# Noninvasive Differentiation of Meningiomas and Dural Metastases Using Intratumoral Vascularity Obtained by Arterial Spin Labeling

**DOI:** 10.1007/s00062-019-00808-x

**Published:** 2019-06-26

**Authors:** Julia Furtner, Isabelle Oth, Veronika Schöpf, Karl-Heinz Nenning, Ulrika Asenbaum, Adelheid Wöhrer, Ramona Woitek, Georg Widhalm, Barbara Kiesel, Anna S. Berghoff, Johannes A. Hainfellner, Matthias Preusser, Daniela Prayer

**Affiliations:** 1 Department of Biomedical Imaging and Image-guided Therapy, Central Nervous System Tumor Unit (CCC-CNS), Medical University of Vienna, Währinger Gürtel 18–20, 1090 Vienna, Austria; 2 Comprehensive Cancer Center, Medical University of Vienna, Währinger Gürtel 18–20, 1090 Vienna, Austria; 3 Institute of Psychology, University of Graz, Universitätsplatz 2, 8010 Graz, Austria; 4 BioTechMed, Mozartgasse 12, 8010 Graz, Austria; 5 Institute of Neurology, Medical University of Vienna, Währinger Gürtel 18–20, 1090 Vienna, Austria; 6 Department of Neurosurgery, Medical University of Vienna, Währinger Gürtel 18–20, 1090 Vienna, Austria; 7 Department of Medicine I, Medical University of Vienna, Währinger Gürtel 18–20, 1090 Vienna, Austria

**Keywords:** Meningioma, Brain metastases, Extra-axial brain tumors, Pulsed arterial spin labeling, Intratumoral neovascularization

## Abstract

**Purpose:**

Using conventional magnetic resonance imaging (MRI) techniques, the imaging features of meningiomas and dural metastases overlap and a differentiation between these tumor entities therefore remains difficult, particularly in patients with a known primary neoplasm. The purpose of this study was to explore the potential role of normalized vascular intratumoral signal intensity values (nVITS) obtained from pulsed arterial spin labeling (PASL) to differentiate between meningiomas and dural metastases.

**Methods:**

In this study PASL was performed in 46 patients with meningiomas (*n* = 30) and dural metastases (*n* = 16) on a 3T scanner, in addition to the routine diagnostic imaging protocol. The ratio between the vascular signal intensity of the tumor and the contralateral normal white matter obtained by PASL images was defined as nVITS.

**Results:**

Meningiomas showed significantly higher nVITS values compared to dural metastases (*p* < 0.001). The optimal nVITS cut-off value to differentiate between the 2 tumor entities was 1.989, with 100% sensitivity and 81.2% specificity.

**Conclusion:**

The nVITS values obtained by PASL provide a fast and noninvasive MRI technique with which to differentiate between meningiomas and dural metastases in a routine clinical setting based on tumor vascularity.

## Introduction

Meningiomas are the most common primary intracranial tumors in adults, representing approximately one third of all intracranial neoplasms [1, 2]. They arise from meningothelial cells of the arachnoid and present as avid, homogeneous, contrast-enhancing dural masses on computed tomography (CT) and magnetic resonance imaging (MRI) [3]. Dural metastases occur much less frequently, representing approximately 1% of intracranial tumors and approximately 10% of patients who suffer from advanced systemic cancer [4]. The most common primary neoplasm from which dural metastases develop are breast, prostate, kidney, lung and head and neck carcinomas [5]. A number of cases have been documented in the literature in which dural metastases were misdiagnosed as meningiomas on conventional imaging [6–8]; however, accurate preoperative differentiation between meningiomas and dural metastases is crucial because this determines a patient’s prognosis and directly influences treatment options [9, 10]. With conventional MRI methods, the differentiation between meningiomas and dural metastases remains difficult because MRI findings overlap in these tumor entities [11, 12]. Previously published findings have already confirmed the advantages of advanced imaging methods in increasing the diagnostic accuracy of meningiomas and dural metastases. In particular, dynamic susceptibility contrast MRI, a contrast-enhanced MRI perfusion technique, has been revealed to be useful in the differentiation between these two tumor types [13, 14]. This technique is, among others, focused on the assessment of perfusion in intra-axial and extra-axial brain tumors; however, in patients with previous allergic reactions to contrast media or in patients with considerably elevated serum creatinine values, the administration of contrast media is critical.

Another MRI sequence used to investigate tumor vascularization is the arterial spin labeling (ASL) technique. This assesses cerebral blood flow (CBF) by using magnetically labeled water protons of the arterial blood that serve as an endogenous tracer. Water protons of the arterial blood are labeled in a labeling slab proximal to the tissue of interest (which is referred to as the imaging volume). Subsequently, the labeled blood reaches the imaging volume where the labeled images (including the delivered magnetization of the arterial blood) are acquired after a predefined inversion time. The perfusion contrast derived by this method comes from subtracting the labeled images from control images (depicting the same tissue of interest without prior labeling of the arterial water protons). Thus, there is no need for the administration of exogenous contrast agents. Using very low inversion times, ASL has been shown to generate normalized vascular intratumoral signal intensity values (nVITS), which correlate with glioma grade and can be used to differentiate between primary cerebral lymphoma and glioblastoma [15, 16].

The purpose of this study was to investigate the utility of nVITS values obtained from ASL to distinguish between meningiomas and dural metastases. This could result in a completely noninvasive method with which to differentiate these meningeal tumor entities.

## Methods

### Patients

Written informed consent was obtained from all patients after the nature, scope, and possible consequences of the examination had been explained. The study was approved by the local institutional review board and performed in accordance with the current guidelines of the Declaration of Helsinki. Histopathological diagnoses were established in all meningioma patients and in 11 (69%) brain metastasis patients by an experienced neuropathologist (A.W.) according to the current criteria of the World Health Organization (WHO) classification system of tumors of the central nervous system [17]. In 5 (31%) brain metastasis patients no tissue specimens were available because they did not undergo surgery or stereotactic biopsy due to a poor general health condition; however, due to the known neoplastic disease, the multiple intracranial and extracranial metastases, and further anatomical imaging details (contrast-enhancing lesion with broad dural attachment that was assumed to be located at least partially extra-axially), these lesions were presumed to be dural metastases and these five patients were included in this subgroup.

### Imaging

The MRI examinations were performed on a 3T MR scanner (Trio Tim, Siemens Medical Solutions, Erlangen, Germany). The conventional MR examination consisted of a coronal T2-weighted turbo spin-echo sequence (TR = 4290 ms; TE 115 ms; flip angle = 120°; number of slices = 56; voxel size = 0.6 × 0.6 × 3 mm), an axial T2-weighted turbo-inversion recovery magnitude (TIRM) sequence (TR = 9220 ms; TE = 100 ms; flip angle = 150°; number of slices = 36; voxel size = 0.9 × 0.9 × 0.4 mm) and a sagittal T1-weighted (magnetization-prepared rapid acquisition with gradient echo, MPRAGE) sequence (TR = 1800 ms; TE = 3.79 ms; flip angle = 12°; number of slices = 192; voxel size = 0.5 × 0.5 × 1 mm; inversion time 1100 ms) with pre-intravenous and post-intravenous contrast medium administration (0.1 mmol/kg body weight of a gadolinium-based contrast agent). In addition to the conventional MRI examination, each patient underwent scanning with a pulsed arterial spin labeling (PASL) sequence. The PASL sequence is a quantitative imaging of perfusion using a single subtraction with interleaved, thin-slice, TI_1_ periodic saturation (Q2TIPS) technique, with a proximal inversion and a control for off-resonance effects (PICORE) tagging scheme [18, 19]. Imaging parameters for the multislice echo-planar imaging PASL sequence were as follows: TR = 2750 ms; TE = 11 ms; voxel size = 3 × 3 × 6 mm; matrix = 64 × 64 mm; number of slices = 14; slice gap = 1.5 mm; flip angle = 90°and number of measurement repetitions = 25. No crusher gradients were used. Based on previously published data, an inversion time of 370 ms was defined [15, 16]. The acquisition time of the PASL sequence was 1 min and 19 s, resulting in an overall acquisition time of approximately 25 min for the whole MRI examination. The PASL sequence was, without exception, acquired before the contrast agent was administered to avoid the T1 shortening effect of gadolinium-based contrast media, which results in a reduction of the signal-to-noise ratio [20].

### Data Analysis

The MRI data were transferred to an off-line workstation (Siemens Leonardo workplace, Erlangen, Germany) for data analysis. In the case of PASL images the automatically generated semiquantitative relative CBF (rCBF) maps of the MR scanner were used to obtain the nVITS values; however, due to the selected short inversion time used in this clinical trial the signal intensity values on the rCBF map do not represent cerebral perfusion values but the obtained signal intensity values can even be negative. Therefore, the scale of the rCBF map has been shifted in order to only contain positive values before calculating the nVITS values.

Conventional MR images were used to define tumor localization and extent. The PASL and contrast-enhanced T1-weighted images were automatically co-registered and if necessary the co-registration was manually corrected. Tumor segmentation was done by an experienced neuroradiologist (J.F.), who was blinded to the tumor histopathology, on the contrast-enhanced T1-weighted images by manually drawing tumor regions of interest (ROIs) slice by slice to approximate to the whole tumor volume. Intratumoral calcifications or macrohemorrhages were attempted to be spared. The selected ROIs were transferred to the rCBF maps obtained by PASL imaging to assess the VITS intensity values as previously described in the literature [15, 16]. The average vascular signal intensity of the whole tumor was calculated by the mean signal intensity values of each slice of the tumor. Another ROI was positioned in the contralateral healthy hemisphere at the level of the centrum semi-ovale in each patient to assess the vascular signal intensity of the normal appearing white matter. The nVITS values were calculated by dividing the average VITS intensity values and the vascular signal intensity values of the normal appearing white matter.

### Statistical Analysis

Sex, age, and normal appearing white matter signal intensity values, were compared for both patient groups using an independent sample t‑test. The nVITS values were compared for both patient groups using a Mann-Whitney U-test. An optimal cut-off value that could produce the sensitivity and specificity required to differentiate meningiomas from dural metastases was determined by a receiver operator characteristics (ROC) analysis. The area under the ROC curve (AUC) values were calculated for nVITS values. Statistical analyses were performed using the Statistical Package for the Social Sciences, Version 24.0 (SPSS, Chicago, Ill, USA). The alpha level for all tests was set at *p* = 0.05.

## Results

A total of 30 meningioma patients (WHO I, *n* = 25; WHO II, *n* = 5) and 16 patients with dural metastases (breast cancer, *n* = 5; lung cancer, *n* = 6; prostate cancer, *n* = 2; renal cancer, *n* = 1; gastric cancer, *n* = 1; melanoma, *n* = 1) were consecutively included in this prospective study. Descriptive statistics about patient characteristics and tumor volume are shown in Table 1.

**Table 1:** Descriptive statistics about patient characteristics and tumor volume

	Meningiomas	Dural metastases
Number of patients (%)	30 (65)	16 (35)
Sex ratio (women:men)	20:10	10:6
Age, median years (range)	61 (37–90)	61 (51–73)
Tumor volume, cm^3^ (range)	35.1 (2–127.9)	18.9 (2.8–80.7)

**Fig. 1:**
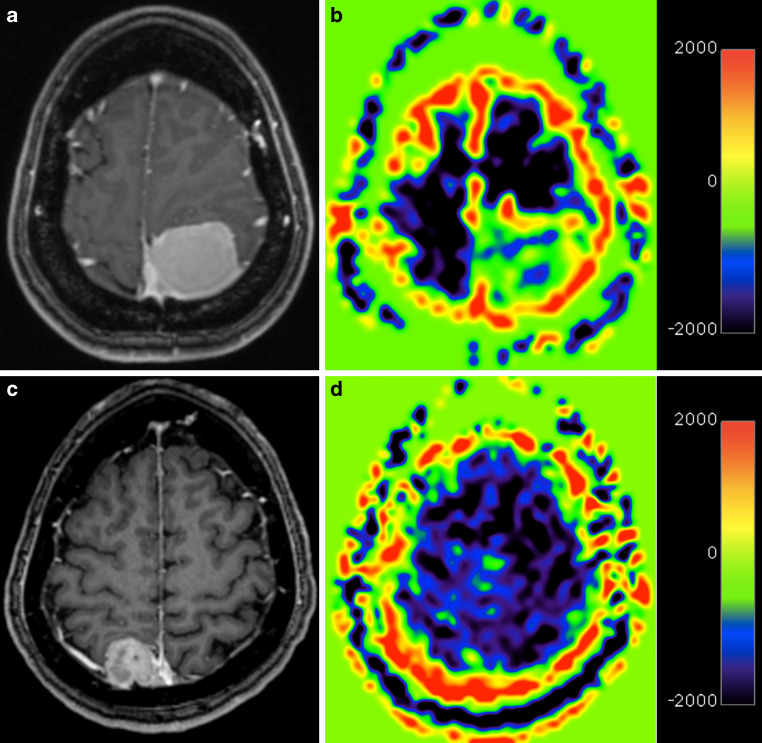
T1-weighted post-contrast images (**a**, **c**) and relative cerebral blood flow maps obtained by pulsed arterial spin labeling (**b**, **d**) of a 41-year-old patient with a meningothelial meningioma (**a**, **b**) and a 52-year-old patient with a dural metastases from breast cancer (**c**, **d**)

Examples of T1-weighted post-contrast images and rCBF maps obtained by PASL of a patient with a meningioma and a breast cancer patient with dural metastases are shown in Fig. 1a–d.

**Fig. 2:**
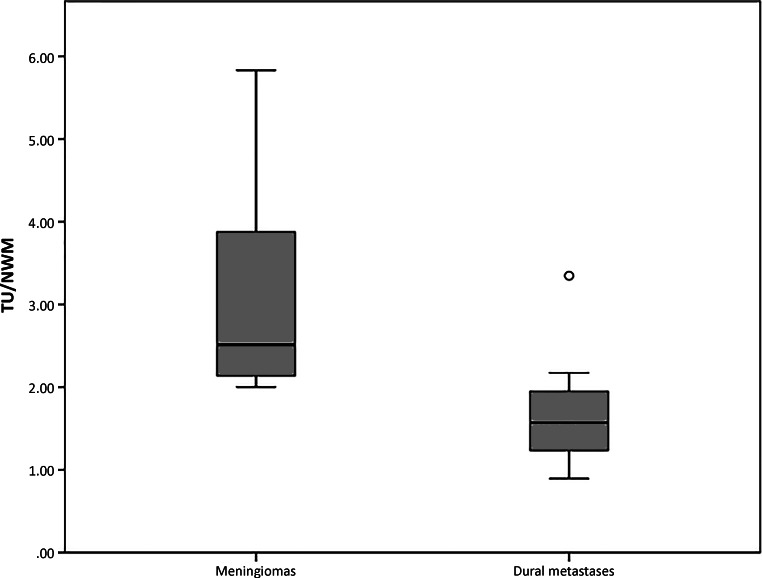
Box-and-whisker plots of normalized vascular intratumoral signal intensity values for meningiomas and dural metastases. Mean normalized vascular intratumoral signal intensity values were significantly higher in meningiomas than in dural metastases (*p* < 0.001). The y-axis depicts the ratio of the signal intensities on the pulsed arterial spin labeling maps between the intratumoral (TU) and the normal white matter tissue (NWM) representing the normalized vascular intratumoral signal intensity values

The mean nVITS value was 3.0 (SD: 1.2, range: 2–5.8) for meningiomas and 1.7 (SD: 0.6, range: 0.9–3.3) for dural metastases. Thus, meningiomas had a significantly higher mean nVITS value than dural metastases (*p* < 0.001). For illustration, see also the box-and-whisker plots in Fig. 2.

**Fig. 3:**
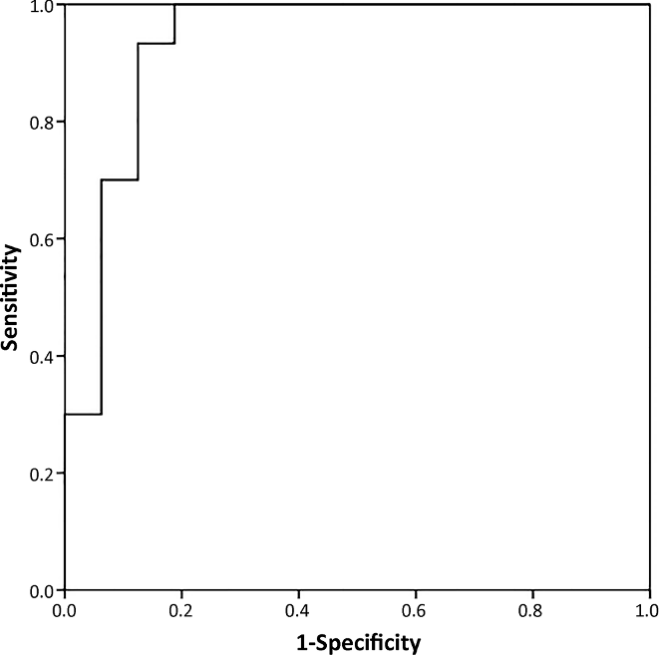
The receiver operator characteristics curves analyzing sensitivity and specificity of normalized vascular intraumoral signal intensity values (nVITS) for differentiating meningiomas and dural metastases show the optimal cut-off value as 1.99 (100% sensitivity and 81.2% specificity)

The ROC curve plotted in Fig. 3 demonstrates that the optimal cut-off value was 1.99 for 100% sensitivity and 81.2% specificity. The AUC value was 0.93.

There were no significant differences in sex (*p* = 0.78), age (*p* = 0.95) and normal appearing white matter vascular signal intensities (*p* = 0.62) between meningioma and dural metastases patients. Moreover, there was no significant difference in the tumor volume (*p* = 0.19); however, there was a wide range of the tumor volume within each tumor entity subgroup (125.9 cm^3^ in meningiomas and 77.9 cm^3^ in dural metastases, respectively).

## Discussion

To determine the most effective treatment option it is of critical importance to be able to differentiate dural metastases from other extra-axial neoplasms that may mimic the characteristic imaging features of this entity. One of the most frequently occurring differential diagnoses includes meningiomas. The features of meningiomas and dural metastases overlap on conventional imaging. Both are characterized as an extra-axial mass, which is isointense to grey matter on T1-weighted images and shows vivid contrast enhancement on T1-weighted, post-contrast images, including the dura, which is called the dural tail sign [11, 12]; however, contrast enhancement represents the disruption of the blood brain barrier rather than tumor neovascularization, which is known to be markedly intensified in meningiomas in contrast to the most common dural metastases, particularly those that derive from a primary breast, prostate or lung carcinoma [13, 21].

The results of this study show that PASL can provide useful information that can differentiate between meningiomas and dural metastases based on tumor vascularity. It was demonstrated that meningiomas show significantly higher nVITS values (*p* < 0.001) compared to dural metastases and a nVITS cut-off value of 1.99 was determined. A nVITS value of 1.99 or less suggested dural metastases, whereas a nVITS value greater than 1.99 suggested meningioma, with a sensitivity of 100% and a specificity of 81%. Therefore, especially in patients with a known primary neoplasm or in the case of an atypically presenting meningioma (e.g. with an adjacent osseous destruction or leptomeningeal extension) the addition of an ASL sequence to the conventional MRI protocol in the routine clinical setting is recommended. Although meningiomas are the most common primary intracranial tumors in adults, a dural lesion with low nVITS values should raise the suspicion of dural metastases; however, in the case of highly vascularized brain metastases, which were represented in this study by one renal cell carcinoma patient, one melanoma patient and one breast cancer patient, nVITS values were increased and overlapped with those of meningioma patients. For this purpose, an additional proton MR spectroscopy to assess an existing alanine peak could help to distinguish highly vascularized brain metastases from meningiomas [22].

The results of this study are consistent with the previously published literature. Kremer et al. [13] and Zimny and Sasiadek [14] revealed that relative cerebral blood volume (rCBV) values obtained by dynamic susceptibility contrast MRI can provide useful information with which to differentiate between dural metastases and meningiomas due to the highly vascular nature of meningiomas compared to the most common dural metastases; however, Kremer et al. also pointed out that rCBV values of hypervascular metastases may overlap with those of meningiomas [23].

To generate perfusion maps obtained by PASL imaging, a postlabeling delay of at least 2 s is recommended. This has been shown to be the time the labeled blood needs to completely enter the tissue space [24]. In contrast, nVITS values are obtained with a lower postlabeling delay time of 370 ms, and therefore, represent tumor vascularization rather than perfusion because the magnetically labeled water protons are primarily intravascular [24]. This technique has recently been revealed to be a useful tool for the differentiation of low-grade and high-grade astrocytomas, as well as of glioblastomas and primary central nervous system (CNS) lymphomas [15, 16]. In this study, it could also be shown that nVITS values offer the possibility to noninvasively differentiate between meningiomas and dural metastases. Therefore, the application of gadolinium-based contrast agents could be avoided, which is an advantage particularly for patients with elevated serum creatinine values or known previously allergic reaction after contrast medium administration. Another advantage of using nVITS values obtained by PASL is the low acquisition time of this MR sequence of 1 min and 20 s. This acceleration, compared to other ASL sequences, where the acquisition times usually range between 3–6 min, can be achieved due to the reduced number of measurement repetitions because of the signal intensification of intravascular blood flow compared with perfusion blood flow within the tumor tissue [24–26]. A negative side effect of the reduced number of measurement repetition is that this is accompanied by a decreased signal to noise ratio; however, because of the extended MR protocols for brain tumors due to advanced MR techniques, the time for each MR sequence should be as short as possible in order to gain the greatest amount of imaging tumor characteristics in the shortest possible time, in terms of patient care. Moreover, a cut-off value for nVITS values (1.99) has been determined, proposing a fast and easily applicable marker in the routine clinical setting with which to differentiate between meningiomas and dural metastases.

A potential limitation of this study is that the calculation of the cut-off value was based on a small sample size. In particular, the number of patients with dural metastases is low due to the relatively rare occurrence of this tumor entity compared to meningiomas [1, 27]. Further studies with an increased sample size as well as different MRI scanner or ASL sequence parameters are needed to support these findings and to take possible variability due to different sequence parameters, MR scanners, manufacturers and postprocessing techniques into account. Moreover, in 5 (31%) brain metastasis patients no tissue specimens were available, because they did not undergo surgery or stereotactic biopsy due to the poor general condition; however, due to the known neoplastic disease, the multiple intracranial and extracranial metastases and the further anatomical imaging details (contrast-enhancing lesion with broad dural attachment that was assumed to be located at least partially extra-axially), those lesions were included in the dural metastases subgroup. Another technical limitation is the limited spatial resolution of the ASL technique. In addition, a voxel size of 3 × 3 × 6 mm and a 1.5 mm spacing were used in this study, which could lead to an underestimation of the signal intensity due to partial volume artefacts especially in small tumorous lesions. The smallest lesion included in this study was a meningioma with a volume of 2 cm^3^, which showed clearly increased VITS intensity values in comparison to the contralateral normal white matter tissue (NWM) (nVITS value = 2.1); however, further studies are needed to investigate also smaller tumorous lesions using this MR perfusion technique. Furthermore, the tumor vascular blood flow may be underestimated in cases of arterial stenosis, which would result in a prolonged bolus arrival time. In order to minimize this potential bias, flow-void signals obtained from T2-weighted images were used to exclude vascular occlusion or profound stenosis; however, non-contrast medium-based vessel imaging techniques, such as time-of-flight angiography, should be added to the MR imaging protocol to rule out marginal reduction of arterial blood flow, which could affect PASL data.

## Conclusion

The nVITS values obtained from PASL sequences noninvasively differentiate between meningiomas and dural metastases on the basis of tumor vascularization with high sensitivity and specificity. Therefore, a dural lesion with low nVITS values strongly argues against the diagnosis of a meningioma and dural metastases should be considered as part of the differential diagnosis. The ASL technique represents a fast and easily applicable MRI technique and in our opinion should be included in the clinical routine MRI protocol for all intracranial neoplasms, including extra-axial tumors.

## References

[1] Wöhrer A, Waldhör T, Heinzl H, Hackl M, Feichtigner J, Gruber-Mösenbacher U, Kiefer A, Maier H, Motz R, Reiner-Concin A, Richtling B, Idriceanu C, Scarpatetti M, Sedivy R, Bankl HC, Stieglbauer W, Preusser M, Rössler K, Hainfellner JA (2009). The Austrian Brain Tumour Registry: a cooperative way to establish a population-based brain tumour registry. J Neurooncol.

[2] Ostrom QT, Gittleman H, Liao P, Vecchione-Koval T, Wolinsky Y, Kruchko C, Barnholtz-Sloan JS (2015). CBTRUS statistical report: primary brain and central nervous system tumors diagnosed in the United States in 2010–2014. Neuro Oncol.

[3] Watts J, Box G, Galvin A, Brotchie P, Trost N, Sutherland T (2014). Magnetic resonance imaging of meningiomas: a pictorial review. Insights Imaging.

[4] Meyer PC, Reah TG (1953). Secondary neoplasms of the central nervous system and meninges. Br J Cancer.

[5] Nayak L, Abrey LE, Iwamoto FM (2009). Intracranial dural metastases. Cancer.

[6] Laidlaw JD, Kumar A, Chan A (2004). Dural metastases mimicking meningioma. Case report and review of the literature. J Clin Neurosci.

[7] Bradley LH, Burton M, Gokden M, Serletis D (2015). Prostate carcinoma mimicking a sphenoid wing meningioma. Int J Surg Case Rep.

[8] Portocarrero-Ortiz L, Garcia-Lopez R, Romero-Vargas S, Padilla JA, Gomez-Amador JL, Salinas-Lara C, Tena-Suck ML, Gonzalez AS (2009). Thyroid follicular carcinoma presenting as skull and dural metastasis mimicking a meningioma: a case report. J Neurooncol.

[9] Goldbrunner R, Minniti G, Preusser M, Jenkinson MD, Sallabanda K, Houdart E, von Deimling A, Stavrinou P, Lefranc F, Lund-Johansen M, Moval EC, Brandsma D, Henriksson R, Soffietti R, Weller M (2016). EANO guidelines for the diagnosis and treatment of meningiomas. Lancet Oncol.

[10] LeRhun E, Weller M, Brandsma D, Van den Bent M, de Azambuja E, Henriksson R, Boulanger T, Peters S, Watts C, Wick W, Wesseling P, Rudà R, Preusser M (2017). EANO Executive Board and ESMO Guidelines Committee. Ann Oncol.

[11] Klingelhöfer L, Mucha D, Geiger K, Koch R, von Kummer R (2015). Prognostic value of conventional magnetic resonance imaging for adult patients with brain tumors. Clin. Neuroradiol..

[12] Zakhari N, Torres C, Castillo M, Nguyen TB (2017). Uncommon cranial menigioma: key imaging features on conventional and advanced imaging. Clin Neuroradiol.

[13] Kremer S, Grand S, Rémy C, Pasquier B, Benabid AL, Bracard S, Le Bas JF (2004). Contribution of dynamic contrast MR imaging to the differentiation between dural metastasis and meningioma. Neuroradiology.

[14] Zimny A, Sasiadek M (2011). Contribution of perfusion-weighted magnetic resonance imaging in the differentiation of meningiomas and other extra-axial tumors: Case reports and literature review. J Neurooncol.

[15] Furtner J, Schöpf V, Preusser M, Asenbaum U, Woitek R, Hainnfellner JA, Wolfsberger S, Prayer D (2014). Non-invasive assessment of intratumoral vascularity using arterial spin labeling: A comparison to susceptibility-weighted imaging for the differentiation of primary cerebral lymphoma and glioblastoma. Eur J Radiol.

[16] Furtner J, Schöpf V, Schewzow K, Kasprian G, Weber M, Woitek R, Asenbaum U, Preusser M, Marosi C, Hainfellner JA, Widhalm G, Wolfsberger S, Prayer D (2013). Arterial spin-labeling assessment of normalized vascular Intratumoral signal intensity as a predictor of histologic grade of astrocytic neoplasms. AJNR Am. J. Neuroradiol..

[17] Louis DN, Perry A, Reifenberger G, von Deimling A, Figarella-Branger D, Cavenee WK, Ohgaki H, Wiestler OD, Kleihues P, Ellison DW (2016). The 2016 World Health Organization Classification of Tumors of the Central Nervous System: a summary. Acta Neuropathol.

[18] Luh WM, Wong EC, Bandettini PA, Hyde JS (1999). QUIPSS II with thin-slice TI1 periodic saturation: a method for improving accuracy of quantitative perfusion imaging using pulsed arterial spin labeling. Magn Reson Med.

[19] Wong EC, Buxton RB, Frank LR (1997). Implementation of quantitative perfusion imaging techniques for functional brain mapping using pulsed arterial spin labeling. NMR Biomed..

[20] Deibler AR, Pollock JM, Kraft RA, Tan H, Burdette JH, Maldjian JA (2008). Arterial spin-labeling in routine clinical practice, part 1: technique and artifacts. AJNR Am. J. Neuroradiol..

[21] Maeda M, Itoh S, Kimura H, Iwasaki T, Hayashi N, Yamamoto K, Ishii Y, Kubota T (1994). Vascularity of meningiomas and neuromas: assessment with dynamic susceptibility-contrast MR imaging. AJR Am. J. Roentgenol..

[22] Majos C, Alonso J, Aguilera C, Serrallonga M, Perez-Martin J, Acebes JJ, Arus C, Gili J (2003). Proton magnetic resonance spectroscopy((1)H MRS) of brain tumours: assessment of differences between tumour types and its applicablility in brain tumour categorization. Eur Radiol.

[23] Kremer S, Grand S, Berger F, Hoffmann D, Pasquier B, Remy C, Benabid AL, Bas JF (2003). Dynamic contrast-enhanced MRI: Differentiating melanoma and renal carcinoma metastases from high-grade astrocytomas and other metastases. Neuroradiology.

[24] Liu P, Uh J, Lu H (2011). Determination of spin compartment in arterial spin labeling MRI. Magn Reson Med.

[25] Kim MJ, Kim HS, Kim J-H, Cho K-G, Kim SY (2008). Diagnostic accuracy and interobserver variability of pulsed arterial spin labeling for glioma grading. Acta Radiol.

[26] Hirai T, Kitajima M, Nakamura H, Okuda T, Sasao A, Shigematsu Y, Utsunomiya D, Oda S, Uetani H, Morioka M, Yamashita Y (2011). Quantitative blood flow measurements in gliomas using arterial spin-labeling at 3T : intermodality agreement and inter- and intraobserver reproducibility study. Ajnr Am J Neuroradiol.

[27] Surawicz TS, McCarthy BJ, Kupelian V, Jukich PJ, Bruner JM, Davis FG (1999). Descriptive epidemiology of primary brain and CNS tumors: results from the Central Brain Tumor Registry of the United States, 1990–1994. Neurooncology.

